# Cell type-specific roles of NLRP3, inflammasome-dependent and -independent, in host defense, sterile necroinflammation, tissue repair, and fibrosis

**DOI:** 10.3389/fimmu.2023.1214289

**Published:** 2023-07-25

**Authors:** Tamisa Seeko Bandeira Honda, John Ku, Hans-Joachim Anders

**Affiliations:** Division of Nephrology, Department of Medicine IV, Ludwig-Maximilians-University Hospital Munich, Munich, Germany

**Keywords:** inflammation, infection, regulated necrosis, innate immunity, interleukin

## Abstract

The NLRP3 inflammasome transforms a wide variety of infectious and non-infectious danger signals that activate pro-inflammatory caspases, which promote the secretion of IL-1β and IL-18, and pyroptosis, a pro-inflammatory form of cell necrosis. Most published evidence documents the presence and importance of the NLRP3 inflammasome in monocytes, macrophages, and neutrophils during host defense and sterile forms of inflammation. In contrast, in numerous unbiased data sets, NLRP3 inflammasome-related transcripts are absent in non-immune cells. However, an increasing number of studies report the presence and functionality of the NLRP3 inflammasome in almost every cell type. Here, we take a closer look at the reported cell type-specific expression of the NLRP3 inflammasome components, review the reported inflammasome-dependent and -independent functions, and discuss possible explanations for this discrepancy.

## Introduction

The nucleotide-binding oligomerization domain (NOD) - and leucine-rich repeat (LRR)-containing receptor pyrin domain-containing (NLRP)-3 have inflammasome-dependent and -independent functions. The NLRP3 inflammasome is a cytosolic pattern recognition platform that integrates infectious and non-infectious danger signals into the secretion of IL-1β and IL-18. The NLRP3 inflammasome is formed by the assembly of NLRP3, the adaptor molecule ASC, and caspase-1, which results in caspase-1 activation, which cleaves pro-IL-1β and pro-IL-18 into their active forms (1). IL-1β and IL-18 can be released actively through gasdermin pores in the plasma membrane or passively during pyroptosis, a type of inflammatory cell death (1, 2). The activation of inflammasomes must be tightly regulated so as to prevent both systemic and tissue inflammation. For example, cryopyrin-associated periodic syndrome (CAPS) results from dysregulated NLRP3 activity due to gain-of-function mutations in *NLRP3*, which leads to abnormal activation of the NLRP3 inflammasome (3, 4). Overproduction of IL-1β has harmful effects in CAPS patients such as hearing loss, urticaria-like rash, joint pain, and inflammation of the eyes, bones, and central nervous system (5, 6). IL-1 antagonists reverse systemic and local inflammation and protect organs in CAPS patients (7–9). Mice carrying the same gain-of-function mutations in the *Nlrp3* gene develop systemic inflammation accompanied by growth retardation and early mortality (10). Conversely, mice lacking *Nlrp3*, or molecules involved in inflammasome assembly, document the deleterious effect of NLRP3 inflammasome activation in sterile inflammation models (11–14).

The vast majority of data on the NLRP3 inflammasome comes from cells of the myeloid lineage. Indeed, single-cell transcriptome data sets consistently report the absence of NLRP3, ASC, caspase-1, and IL-1β transcripts from cell types other than myeloid cells (https://www.proteinatlas.org/). These data are consistent with the absence of IL-1β protein in parenchymal tissues. Cell type-specific modes of immune activation are an important element in limiting potentially harmful inflammation, cytokine storms, and immunopathology. Nevertheless, a surprising number of studies report NLRP3 expression and activity, and even IL-1β secretion, by human and mouse epithelial cells and other parenchymal tissue cells (**Table 1**). This is not supported by many unbiased expression data sets.

**Table 1:** Some experimental studies reporting expression and function of the NLRP3 inflammasome in non-myeloid cells.

Cellular type	NLRP3, ASC Expression	IL-1β Release	IL-18 Release	Pyroptosis	Additional function	Reference
T cells	Yes	Yes		Yes	IFN-γ production	(16–18)
	No	No		No	Th2 phenotype - Transcript factor	(19, 20)
B cells	Yes	Yes			IgM production	(21, 22)
	Yes	Yes			Homing and B cell differentiation	(21–23)
Endothelial cells	Yes	Yes	Yes		Endothelial dysfunction	(24, 25)
	Yes	Yes		Yes	Endothelial dysfunction	(24, 26, 27)
Epithelial cells	Yes	Yes			Foot process effacement, proteinuria	(28–30)
	Yes	No	No		TGFβR signaling	(31)
	Yes	Yes			TGFβR signaling	(32)
	Yes	Yes	Yes			(33–35)
Fibroblast	No	No	No		TGFβR signaling	(36)
	No	No	No		TGFβR signaling, Myofibroblasts differentiation	(37)

IFN-γ (Interferon-gamma), Th2 (T helper 2), IgM (Immunoglobulin M), TGFβR (Transforming grow factor β) receptor).

**Figure 1:**
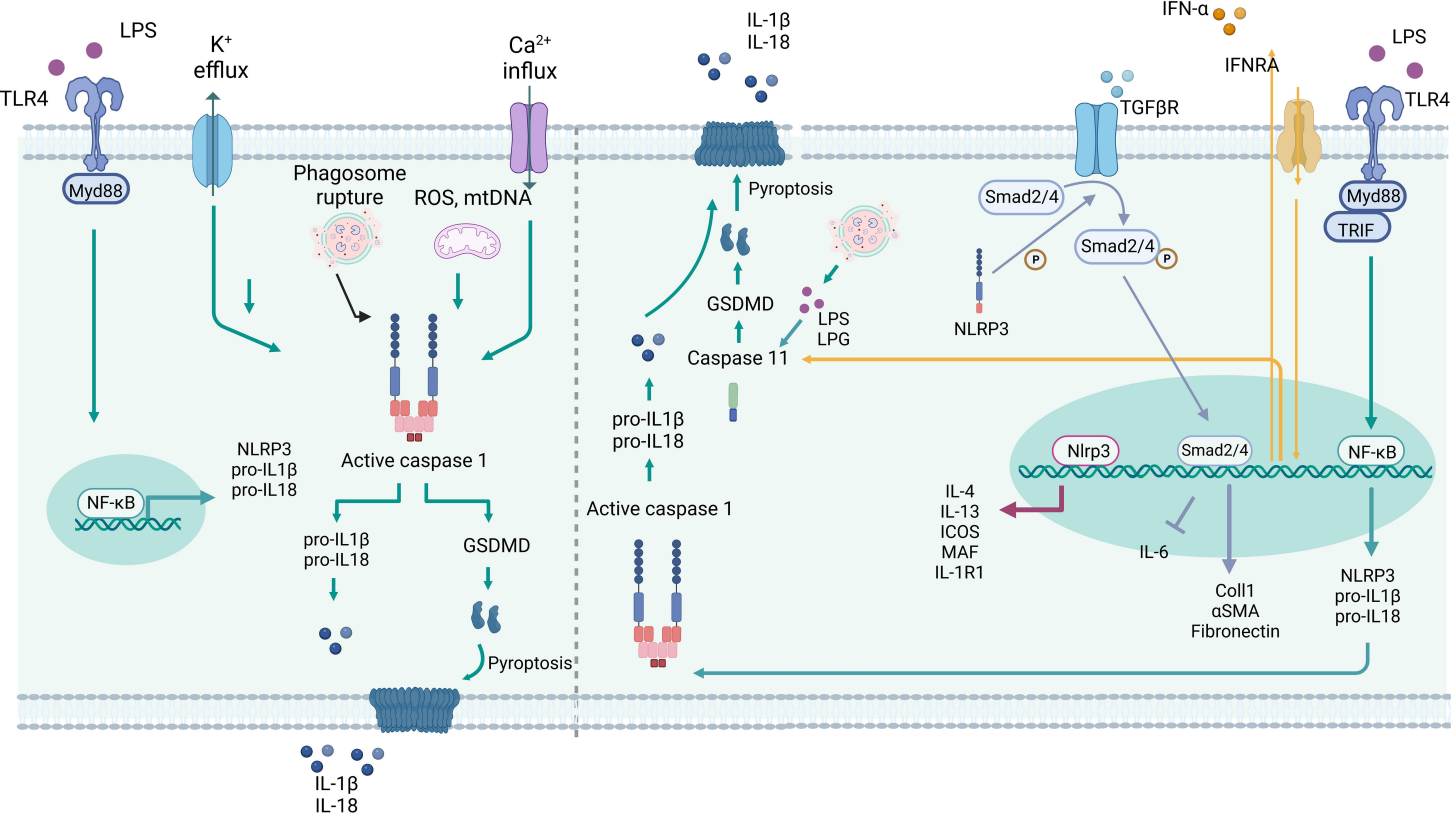
Inflammasome-dependent and –independent roles of NLRP3 in myeloid cells. Left: Canonical activation of NLRP3 starts with TLR activation, mediate by Myd88 and NF-κB, which triggers the expression of NLRP3, pro-IL-1β and -IL-18. Several stimuli can trigger the inflammasome assembly, such as K_+_ efflux, Ca_+2_ influx, crystals, phagosome rupture, mtROS and mtDNA. The conjunction of NLRP3, ASC and Caspase 1 lead to Caspase 1 activation and proteolytic cleavage of pro-IL-1β and -IL-18, as well GSDMD resulting in IL-1β and -IL-18 secretion and/or pyroptosis. Right: Non-canonical NLRP3 activation is triggered by TLR activation, via Myd88 and TRIF, or type I interferon receptor (IFRA), which lead to NF-κB activation and induction of NLRP3, pro-IL-1β and -IL-18 expression. The type I interferon triggers caspase-11, and caspase 1 expression. LPS and LPG can bind and directly activate caspase 11, which cleaves GSDMD in GSDMD-N driving pyroptosis. Nlrp3 can act as a transcription factor in T cells by binding directly to the *Il4*, *Il13*, *Icos*, *Maf*, *Il1r1* promoter region. Nlrp3 can induce phosphorylation of Smad2/4 in the TGFβR pathway, thereby regulating the transcription of target genes. Created with BioRender.com.

Here we review the published data on the inflammasome-dependent and inflammasome-independent roles of NLRP3 to better understand the function of NLRP3 in tissue-specific danger signaling and beyond the secretion of IL-1β (**Figure 1**). Furthermore, we call for a critical discussion of the discrepancy between the apparent absence of transcripts of NLRP3 inflammasome components and of many cell types that are reported to carry out NLRP3 inflammasome functions. In addition, we discuss the experimental tools used in this context.

## Inflammasome- versus non-inflammasome-related functions of NLRP3

### Canonical NLRP3 inflammasome signaling

NLRP3 inflammasome activation is preceded by the canonical and non-canonical pathways. Both pathways initiate the induction of inflammasome components, and their substrates, following cytokine or Toll-like receptor (TLR) activation. This increases the transcription of pro-IL-1β, pro-IL-18, and other NLRP3-related molecules *via* the translocation of NF-κB into the nucleus. In the canonical pathway, danger signals promote the oligomerization of NLRP3, the adaptor protein ASC, and pro-caspase-1. Once activated, caspase-1 cleaves pro-IL-1β and pro-IL-18 at aspartate residues to generate the effector forms of these cytokines (15). Furthermore, caspase-1 activation leads to the cleavage of gasdermin D (GSDMD), which can disrupt inner membrane lipids (phosphatidylserine and phosphatidylinositol) and cell osmolality, triggering pyroptosis and releasing alarmins such as IL-1α, IL-1β, and IL-18 (15).

### Non-canonical NLRP3 inflammasome signaling

**Figure 2:**
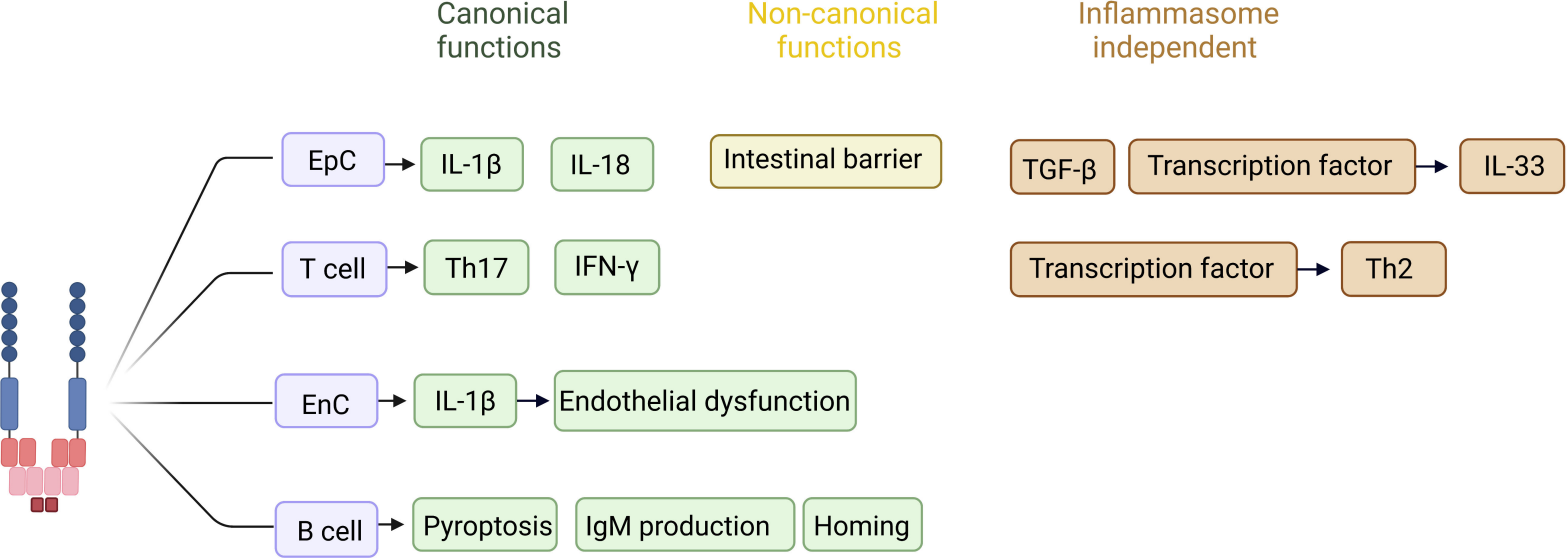
Reported inflammasome-dependent and -independent functions of NLRP3 in non-immune cells. In T cells, canonical activation of NLRP3 has been described to favor differentiation of naive T cells into Th17 cells, as well as to increase IFN-γ production in the face of viral infection. In an inflammasome independent way, the NLRP3 acts as a transcription factor in T cells, favoring a Th2 profile. In epithelial cells, canonical activation of NLRP3 is related to the production and release of IL-1β and IL-18 during inflammatory processes, whereas the non-canonical pathway favors the maintenance of the intestinal epithelial barrier. In epithelial cells the inflammasome independent function is related with TGF-β pathway activation and fibrosis, as well acting as a transcription factor controlling the expression of IL-33. The role of NLRP3 in endothelial cells has been pointed out during inflammatory processes, in which IL-1 β production favors endothelial dysfunction and increased expression of selectins, acting on the recruitment of inflammatory cells to the injured site. In B cells NLRP3 stimulates the IgM production, as well the expression of CXCR4 and CCR7, homing related chemokines. *EpC (epithelial cell). *EnC (endothelial cell). Created with BioRender.com.

Non-canonical NLRP3 inflammasome activation involves activation of caspases 4 and 5 in humans and caspase-11 in mice (15). Prior to this, NF-κB translocates to the nucleus, where it increases the transcription of interferon regulatory factors (IRF) 3 and 7, and induces the expression of IFN-α/β (**Figure 2**). These IFNs trigger the expression of caspase-11, which cannot cleave pro-IL-1β to IL-1β, but through the induction of caspase-1, the active form of IL-1β can be generated, i.e., the non-canonical pathway to induce IL-1β (38). In addition, caspase-11 can be activated by physically binding to LPS (39) or lipophosphoglycan (LPG) (40). Once activated, caspase-11 can also induce pyroptosis by cleaving GSDMD. The main role of the non-canonical NLRP3 is to protect against Gram-negative bacteria that evade the phagosome and invade the cytosol (41). In this way, Cheng et al. showed that LPS can activate lung endothelial cells towards pyroptosis, which is abolished in *Casp11*-deficient endothelial cells (42).

### Inflammasome-independent roles of NLRP3

An inflammasome-independent function of NLRP3 is the promotion of fibrosis through regulation of the TGF-β signaling pathway (31, 32, 37, 43). The absence of *Nlrp3* in epithelial cells, but not caspase 1, IL-1β, or IL-18, reduced their ability to produce αSma and Mmp9, and decreased epithelial-mesenchymal transition when stimulated with TGF-β (31). Numerous studies demonstrate that *Nlrp3* regulates TGF-β through the phosphorylation of Smad2/3 (31, 37, 44–46). Furthermore, NLRP3 acts as a transcription factor in the nucleus of T cells, promoting Th2 polarization through its ability to bind directly to DNA, specifically in the promoter regions of *Il-4*, *Il-13*, *Il1r1*, *Icos*, and *Maf* (19). Similarly, *Nlrp3* acts as a transcription factor and regulates the expression of IL-33 in epithelial cells (47). In addition, *Nlrp3* deficiency or inhibition prevented the production of IL-33 in a model of atopic dermatitis (47). We will discuss in more detail the functions of NLRP3 that are not related to inflammasome activation in specific cell types.

## NLRP3 in monocytes/macrophages

Monocytes and macrophages are mononuclear phagocytes that play a key role in innate immunity and that have distinct roles in tissue homeostasis and immune response (48). Both cell types can recognize pathogen- and damage-associated molecular patterns that trigger inflammation and immunity. Monocytes are circulating cells that differentiate into tissue macrophages upon transmigration into injured tissues, whereas tissue macrophages recognize, phagocytose, and degrade pathogens or cellular debris, contributing to tissue repair and fibrosis (49).

### Canonical NLRP3 inflammasome signaling

In mononuclear phagocytes, a broad spectrum of exogenous stimuli ranging from crystalline microparticles to viral proteins (SARS-CoV viroporin, hepatitis C virus core protein, and influenza virus M2) can activate the NLRP3 inflammasome (50–52). In addition, NLRP3 acts as a sensor of host-derived danger signals and can detect changes in membrane lipids, ion efflux, mitochondrial dysfunction, and the production of reactive oxygen species (ROS) (53, 54). The ability to secrete large amounts of IL-1β and ROS contributes to the defense against pathogens, but the mechanism by which these effector molecules control pathogen load differs from one type of microorganism to the next (55–57) (57–59). The mechanism by which macrophages resist intracellular protozoan infection depends on IL-1β production *via* activation of the NLRP3 inflammasome. Binding to IL-1R and activation of Myd88 signaling trigger NO production (55, 57). The dependence of ROS on NLRP3 inflammasome activation has also been shown in a model of the influenza virus, where treatment of infected macrophages with N-acetyl-L-cysteine (NAc) reduced inflammasome-dependent IL-1β production and, consequently, impaired pathogen control (58).

### Non-canonical NLRP3 inflammasome signaling

NLRP3 activation is associated with a change in macrophage phenotype, a mechanism independent of IL-1β (36, 60–62). Indeed, the lack of silencing of NLRP3 reduces the number of anti-inflammatory macrophages and/or their ability to secrete anti-inflammatory cytokines, such as IL-10 and IL-4 (36, 61). However, NLRP3 inhibition favors macrophage infiltration and/or macrophage reprogramming to a pro-inflammatory phenotype in models of chronic kidney disease (CKD) and pancreatic cancer (62–64) .

The fact that macrophages do not require a priming step to assemble the components of the NLRP3 inflammasome highlights the importance of regulatory mechanisms to avoid chronic and systemic inflammation (65). Macrophage migration inhibitory factor (MIF) regulates NLRP3 inflammasome assembly and activation *via* the inhibition of ASC speck formation and caspase-1 cleavage, rather than by interfering with NF-κB activation (66). Another mechanism of regulation of NLRP3 in macrophages is the methylation of *Cpt1a*, a gene involved in ROS and energy production *via* fatty acid oxidation and oxidative phosphorylation (67).

## NLRP3 in dendritic cells

Dendritic cells are professional antigen-presenting cells due to their ability to induce activation and differentiation of naive T cells (68). DCs upregulate inflammasome components upon a priming signal *via* NF-κB-dependent transcription (69, 70).

### Canonical NLRP3 inflammasome signaling

In DCs, the activation of the NLRP3 inflammasome can lead to pyroptosis or hyperactivation (71). During hyperactivation, DCs produce high amounts of IL-1β while remaining viable and increase their migratory ability to lymph nodes (72, 73). Furthermore, it was observed that through IL-1β production, NLRP3 induces a lowered tolerogenic profile in DCs (74). In addition to its role in the activation of DCs, IL-1β secretion is essential for the priming of naive T cells. DCs lacking *Casp1* fail to activate CD8^+^ T cells (75).

### Inflammasome-independent roles of NLRP3

In different pathological models, activation of the NLRP3 inflammasome in DCs can have deleterious effects. Mice lacking *Nlrp3* or by gene inhibition, have been shown to favor DCs toward a tolerogenic phenotype, leading to a better disease outcome (74, 76–78). This is in surprising contrast to the role of NLRP3 in a model of lupus-like systemic autoimmunity, where the absence of *Nlrp3* and *Casp1* accelerated the systemic autoimmune process; NLRP3 is needed to maintain the immunosuppressive effect of TGFβR signaling that counterbalances adaptive immunity (45). In this disease context, NLRP3 contributes to the generation of suppressor DCs (79). Indeed, *NLRP3* ^-/-^ and *Casp1* ^-/-^ DCs show less Smad2/4 phosphorylation, which is required in the TGFβR signaling pathway to suppress the expression of pro-inflammatory cytokines such as IL-6 (44, 45).

## NLRP3 in granulocytes

### Neutrophils

Neutrophils are the most abundant granulocyte in blood circulation and are the fastest line of defense against bacterial and fungal infections (80). When confronted with a microorganism, neutrophils can release antimicrobial proteins from their granules, ROS from their cytosol, and extracellular traps (NETs) to entrap pathogens (81). The same effector mechanisms of neutrophils are involved in sterile inflammation (82).

Knowledge about NLRP3 and neutrophils is not as well established as knowledge about macrophages and dendritic cells. Neutrophils possess the machinery required for IL-1β production, in an NLRP3 inflammasome-dependent manner in response to *Staphylococcus aureus* infection (83, 84). Furthermore, neutrophils carrying gain-of-function mutations in *Nlrp3* have been observed to increase cytoplasmic granule exocytosis as well as NETosis capacity (85, 86).

#### Canonical NLRP3 inflammasome signaling

In neutrophils, NLRP3 positively regulates their recruitment to an inflammatory site through IL-1β production. Once in the absence of *Nlrp3*, neutrophils secrete lower amounts of IL-1β, which in turn decreases the activation of endothelial cells and the expression of P-selectin, which is necessary for neutrophil rolling and transmigration (87).

In a model of *Streptococcus pneumonieae* infection, tumor necrosis factor-α (TNF-α) and pneumolysin toxin can trigger IL-1β production through NLRP3 inflammasome activation. IL-1β production is required to activate γδT17 cells and, consequently, IL-17 production, which supports infection control (88). On the other hand, inhibition of NLRP3 and caspase-1 increased ROS production by neutrophils and their ability to control the levels of uropathogenic *Escherichia coli* (89).

#### Non-canonical NLRP3 inflammasome signaling

The mechanism connecting the NLRP3 inflammasome to NET extrusion or even NETosis is caspase-1-independent. Chen et al. showed in a PAMP-rich milieu, that neutrophils produce IL-1β, but do not undergo caspase-1-dependent pyroptosis, due to the inability of caspase-1 to cleave the GSDMD pore-forming p30 fragment (90, 91). On the other hand, caspase-11 is more efficient at cleaving GSDMD in neutrophils. The combination of high caspase-11 expression and cleaved GSDMD is necessary for neutrophil plasma membrane rupture and NET extrusion, which acts as a defense mechanism against cytoplasmic microorganisms (90, 92). The requirement for plasma membrane rupture for NET release is still under debate, as it has recently been shown that NETs can be released from viable neutrophils that have not undergone pyroptosis (93). This distinct resistance to pyroptosis may be explained by the ability of neutrophils to resist mitochondrial depolarization, which is a trigger for NLRP3 inflammasome activation (91).

#### Inflammasome-independent roles of NLRP3

In a model of hepatic ischemia/reperfusion, the absence of *Nlrp3* was associated with fewer neutrophil infiltrates, but not in *Asc ^-/-^* or *Casp1^-/-^* mice, which indicates an inflammasome-independent role of *Nlrp3* in neutrophils. Mechanistically, *Nlrp3-*deficient neutrophils are less responsive to chemokines that guide their migration (94).

### Eosinophils

#### Canonical NLRP3 inflammasome signaling

The role of NLRP3 in eosinophils has not been well characterized. In a helminth infection model, Alhallaf et al. observed that the absence of NLRP3 increased the number of eosinophils in mesenteric lymph nodes (95). This phenomenon was also found in allergic mice and could be explained by a microenvironment with high levels of IL-33, IL-13, and IL-5 (96).

### Mast cells

Mast cells are granulocytes found in connective tissues throughout the body. Their localization around blood vessels enables them to regulate vasodilation and vascular homeostasis by secreting angiogenic or vasoactive factors, including vascular endothelial growth factor (VEGF), TNFα, and histamine (97).

#### Canonical NLRP3 inflammasome signaling

Like the other granulocytes, mast cells have functional inflammasome components to activate caspase-1 and induce the secretion of IL-1β. In the skin of CAPS patients, mast cells are the major producers of IL-1β; cell-producers in the skin of CAPS patients are mast cells (98). The ability of mast cells to produce IL-1β has also been demonstrated in a model of endometriosis, in which estrogen-stimulated mast cells can produce IL-1β via K^+^ efflux. Furthermore, it has been observed that the NLRP3 promoter region contains estrogen responsive elements (99). The anti-allergic drug Tranilast can directly inhibit the NLRP3 inflammasome assembly and reduce IL-1β and IL-6 production (100).

## NLRP3 in lymphocytes

Lymphocytes are produced in the bone marrow and mostly patrol and reside in lymphoid tissues, where they can undergo maturation and clonal expansion upon presentation of their cognate antigens as part of antigen-specific adaptive immune responses (16). These lymphocyte lineage cells can be divided into T and B cells, which are part of the adaptive immune system, while NK cells and other innate lymphocytes support innate immunity (16).

According to RNAseq analysis provided by the Human Protein Atlas, T cells express the machinery necessary to assemble the NLRP3 inflammasome (*NLRP3*, *CASP1*, and *PYCARD*), while B cells express only *CASP1* and *PYCARD*.

### T cells

#### Canonical NLRP3 inflammasome signaling

Studies in mice carrying an NLRP3 gain-of-function mutation have shown that hyperactivation of the NLRP3 inflammasome induces differentiation of naive T cells into Th17 cells, in a mechanism supported by high levels of IL-1β (17, 101). The role of NLRP3 in regulating T cell phenotype has been described in HIV patients and models of autoimmune disease (18, 20). In addition, autocrine canonical activation of the NLRP3 inflammasome in CD4^+^ T cells is necessary to enhance IFN-γ production during viral infection (102). During intracellular microorganism infection, the NLRP3 inflammasome-caspase-1 axis induces T cell pyroptosis, which is considered to be the major mechanism associated with T cell depletion (20, 21, 23).

#### Inflammasome-independent roles of NLRP3

In a mechanism independent of IL-1β generation, NLRP3 can regulate T cell subsets by promoting a Th2-profile (22). Due to its nuclear localization in Th2 cells, NLRP3 may act as a transcription factor capable of binding to promoter regions of genes involved in the Th2 signature, such as *IL-4*, *IL-13*, *IL1R1*, *ICOS*, and *MAF* (19, 103).

### B cells

B cells are key regulators of the adaptive humoral immune system and are responsible for the production of immunoglobulins directed against pathogen-related antigens (104). Unlike T cells, which clearly express the NLRP3 inflammasome, RNAseq analysis indicates that B cells express low amounts of *NLRP3*, although they express *ASC* and *CASP1*. B cells are capable of canonically activating the NLRP3 inflammasome and driving IL-1β production upon stimulation with β-glucan, a fungal antigen, or even with B cell activating factor (BAFF) (105, 106).

#### Canonical NLRP3 inflammasome signaling

Although the role of NLRP3 in B cells is not yet fully understood, it is believed to be associated with the maintenance of an inflammatory environment that favors the development, homing, and retention of B cells in lymphoid organs. When stimulated with BAFF (one of the major pro-survival factors involved in B cell homeostasis), human B cells assemble the NLRP3-caspase-1 complex, triggering IL-1β production (107). When activated in the absence of *Nlrp3*, B cells produce lower IgM titers (106). The absence of *Nlrp3* was associated with a lower expression of markers of B cell homing and differentiation, such as CXCR4 and CCR7 (105, 106). However, it has not yet been proven that this function is associated with the activation of the NLRP3 inflammasome.

## NLRP3 in endothelial cells

Endothelial cells form the inner lining of the heart, blood, and lymphatic vessels. In addition to their barrier function, endothelial cells help maintain the immune privilege of certain tissues. Under homeostatic conditions, endothelial cells produce antiplatelet and anticoagulant molecules to prevent platelet aggregation and fibrin formation.

During endothelial dysfunction, endothelial cells express adhesion molecules and chemokines that allow for the recruitment and transmigration of immune cells to the inflamed sites and the upregulation of procoagulant mediators, such as tissue factor and von Willebrand factor (87, 108). Chronic inflammation is considered one of the main causes of endothelial dysfunction (24). IL-1β and ROS, among other inflammatory mediators, can activate endothelial cells and contribute to their dysfunction and are produced by circulating immune cells, e.g., neutrophils (25, 87). The Human Protein Atlas reports transcript expression under basal conditions in endothelial cells from different organs that do not appear to express relevant levels of *NLRP3 mRNA*, although they do express *ASC* and *CASP1*.

### Canonical NLRP3 inflammasome signaling

Diabetes, obesity, atherosclerosis, and stroke are triggers that can activate endothelial cells to increase *NLRP3 expression*, which may contribute to endothelial dysfunction (26, 27, 109). DAMPs (HMGB1 and cold-inducible RNA-binding protein) increase the expression of the NLRP3 inflammasome in endothelial cells (110–112). However, activation of the NLRP3 inflammasome generates high concentrations of IL-1β, which contributes to endothelial injury (111). The cleavage of GSDMD and induction of pyroptosis, by both canonical and non-canonical pathways, have been observed in endothelial cells under pathological conditions (28, 110, 112). Some approved drugs, such as statins, hypoglycemic agents, and anti-inflammatory drugs reduce endothelial injury by inhibiting NLRP3 inflammasome activation (27, 29, 33, 109).

## NLRP3 in epithelial cells

Epithelial cells cover body surfaces and cavities and provide barrier function and transepithelial exchange of fluids, ions, metabolites, and other nanomolecules as part of absorption, excretion, and filtration. The Human Protein Atlas reports that under healthy conditions, epithelial cells from the kidneys, lung, skin, intestine, and other organs lack *NLRP3* expression, which for methodological reasons does not exclude low-level transcripts. NLRP3 inflammasome-related transcripts (*ASC* and *CASP1*) are present in goblet cells, are poorly expressed in keratinocytes, and are not detectable in lung or kidney epithelial cells. However, NLRP3 induction under stress conditions has been demonstrated (30, 34, 35, 113).

### Kidney epithelial cells

#### Canonical NLRP3 inflammasome signaling

Although kidney epithelial cells do not appear to express much of *NLRP3* mRNA under healthy conditions, kidney biopsies from patients with lupus nephritis showed an increase in NLRP3 expression in podocytes (34). The increase in *NLRP3* was accompanied by higher expression of *CASP1* and *ILB*β and correlated positively with higher levels of proteinuria (114). In a similar manner, pharmacological inhibition of Nlrp3 improved kidney function in a model of APOL1-associated podocytopathy through the reduction of *Il1b* and *IL-6* (35). Podocyte-specific depletion of NLRP3 demonstrated a positive role for the NLRP3 inflammasome in protecting podocytes from glomerular pathology in a mouse model of diabetic kidney disease. Evidence also points to NLRP3 exerting a non-canonical effect on podocytes, as the absence of caspase-1 and IL-1β was only partially protective (115).

#### Inflammasome-independent roles of NLRP3

Beyond the classical pro-inflammatory roles of NLRP3, *in vitro* studies with primary tubular epithelial cells (TECs) isolated from *Nlrp3*-deficient mice showed that NLRP3 regulates the TGF-β pathway in an inflammasome-independent manner. Using NLRP3^-/-^ TECs, Wang et al. observed a reduction of EMT markers, such as TGFβ-1, MMP-9, and *ACTA2* after stimulation with TGF-β1 (31). A similar study in lung epithelial cells showed that inhibition of NLRP3 prevented EMT by upregulating *CHD1*, while downregulating TGFβ-1, and *ACTA2*, preventing morphological changes towards a fusiform shape (32). In contrast, anti-GBM glomerulonephritis involves IL-1 but is independent of NLRP3 inflammasome-mediated activation of caspase-1 (116).

### Keratinocytes

Keratinocytes are specialized epithelial cells of the epidermis that constitutively express pro-IL-1β, as a defense mechanism against foreign antigens found in the skin. However, under homeostatic conditions, keratinocytes cannot release the active form of IL-1β (117).

#### Canonical NLRP3 inflammasome signaling

Skin inflammation caused by UVB irradiation induces caspase-1 activation and IL-1β processing by increasing cytoplasmic Ca^2^ (113, 118). Viral dsDNA and self-DNA, released from cells damaged by mechanical insults, UVB inflammation, and/or skin diseases such as psoriasis, can lead to increased NLRP3 activation (119, 120) In a psoriasis model, it was observed that keratinocytes stimulated by IL-17 and IL-22 upregulated *Il1β*, *via* ROS-induced NLRP3 activation (121). The use of drugs that inhibit NLRP3 and/or caspase-1 activation, such as metformin and ginsenoside Rg1, has shown a positive effect in the treatment of psoriasis-like lesions by inhibiting *Il1b* and keratinocyte proliferation (122, 123).

#### Inflammasome-independent roles of NLRP3

Recently, NLRP3 has been shown to localize to the keratinocyte nucleus, act as a transcription factor, and regulate IL-33 expression. Lack of NLRP3 has been shown to reduce IL-33 mRNA and protein levels, thereby improving the lesions associated with topical dermatitis (47).

### Intestinal epithelial cells

Intestinal epithelial cells have pleiotropic functions ranging from hormone-like secretion to regulation of the intestinal microbiota and the host immune system. Maintenance of the epithelial barrier in the intestine is essential to prevent microorganism translocation.

#### Canonical NLRP3 inflammasome signaling

In a model of *Citrobacter rodentium* infection, it was observed that mice lacking *NLRP3* and *Casp1* were more susceptible to bacterial penetration into the intestinal crypts (124). In a model of dextran sodium sulfate colitis, it was observed that *Il1β* played a role in intestinal epithelial repair and epithelial barrier formation. *Il1β* deficiency reduced the proliferation of intestinal epithelial cells and the expression of tight junction proteins while impairing intestinal permeability (125). Similarly, DNA sequencing analysis showed that mutations in the downstream regulatory region of *NLRP3* are associated with lower expression of *Il1β* and consequently increased susceptibility to Crohn’s disease (126). Taken together, these data show that activation of the NLRP3 inflammasome in intestinal epithelial cells plays an important physiological role in maintaining the intestinal barrier and limiting pathogen colonization.

#### Non-canonical NLRP3 inflammasome signaling

Non-canonical activation of NLRP3 *via* activation of caspase-11 positively regulates the integrity of the intestinal epithelium by stimulating intestinal epithelial cell proliferation. In a model of colitis, it was observed that the absence of *Casp11* impaired IL-18 production, increasing susceptibility to colitis (127, 128). Although the function of cytokines related to the activation of the NLRP3 inflammasome in the protection of the intestinal barrier is recognized, its origin seems to be unclear, since it has been observed that in the absence of caspase-11, there is a compensation of caspase-1 expression and the production of IL-1β (129), which has a protective role in intestinal epithelial cells (125).

## NLRP3 in other non-immune cells

Hepatocytes are the main parenchymal cells in the liver involved in detoxification, lipid metabolism, albumin synthesis, and the secretion of coagulation factors. The Human Protein Atlas shows that under homeostatic conditions, hepatocytes do not express *NLRP3* and *ASC*, although they express low levels of *CASP1*. Some studies show that diabetes, liver inflammation, and steatosis induce the expression of *Nlrp3*, *Casp1*, and *Il1b* in the liver tissue, including immune and non-immune cells (46, 124, 125). Activation of the NLRP3 inflammasome by ROS induces the release of IL-1β and IL-18 by hepatocytes, which then undergo pyroptosis (130). Hepatocytes carrying gain-of-function mutations were sufficient to drive spontaneous collagen deposition in the liver (131). Conversely, hepatocytes with *Nlrp3*-deficiency and/or pharmacological inhibition of Nlrp3 ameliorate liver inflammation by reducing IL-1β, IL-6, and TNF-α production; they also ameliorate liver fibrosis by reducing Col1a and aSma. Together, this information shows that canonical inflammasome activation in hepatocytes is sufficient to propagate liver injury and fibrosis.

## Possible explanations for the discrepancy between the absence of inflammasome transcripts and the published evidence in non-immune cell types

There is a discrepancy between transcriptional data obtained from RNAseq analysis and functional studies of the NLRP3 inflammasome in non-immune cells. One of the possible explanations is the low sensitivity of single-cell RNA sequencing analysis, which may miss low levels of transcripts under homeostatic conditions. However, different RNA sequencing data sets and data available on the Human Protein Atlas from certain disease conditions, such as diabetes, ischemia-reperfusion, or tissue remodeling, still do not report NLRP3 inflammasome-related transcripts in epithelial cells (GSE131882, GSE119531, and GSE206084). The most common form of functional evidence is provided by *in vitro* studies employing immortalized cell lines that may have undergone somatic mutations and phenotypic changes over time. In this regard, primary cell cultures would seem to provide more solid evidence. However, such primary isolates are frequently contaminated by tissue-resident immune cells, leading to erroneous conclusions. The use of non-specific antibodies in immunohistochemistry and Western blotting may also be one of the reasons for this discrepancy (132). In fact, less than half of the routinely used antibodies bind to their specific targets (133). Therefore, the specificity of the NLRP3 antibody should be questioned, especially when used as a negative control, when documenting tissue samples as NLRP3-deficient.

The use of constitutive NLRP3 knockout animals has greatly contributed to several discoveries about the canonical role of the NLRP3 inflammasome, mainly in immune cells. However, such animals cannot determine the cell type-specific effects of the NLRP3 inflammasome. Similarly, NLRP3 inhibitors or whole-body knockout mice cannot attribute an improvement of the condition to the sole and exclusive inhibition of the inflammasome in parenchymal cells without considering the participation of immune cells (27, 29, 109, 122). Thus, studies concluding on NLRP3 inflammasome activity in non-immune cells have to be carefully evaluated for methodological consistency and possible involvement of myeloid cells.

The use of cell type-specific knockout mice, e.g., with a floxed NLRP3 motif under the control of cell type-specific expression of Cre recombinase, may help to determine the role of the NLRP3 inflammasome in parenchymal cells. However, such experiments need a series of controls to validate the cell type-specific depletion and exclude Cre leakage or epigenetic silencing of Cre recombinase (134–136). Another approach to evaluating the role of NLRP3 in specific cell types is through tools that induce NLRP3 expression, such as the use of animals with gain-of-function mutations and the transfection of cells with the gene of interest. For example, animals with the human A350V gain-of-function mutation and/or deletion of NLRP3 in podocytes have shown that activation of this inflammasome is sufficient to promote the glomerular damage observed in animals with diabetic kidney disease (115). In a similar manner, the specific deletion of *Nlrp3* in epithelial cells demonstrated the importance of this inflammasome in promoting TGF-β signaling (31).

## Conclusions and perspectives

NLRP3 is involved in multiple cellular mechanisms. The canonical activation of NLRP3 is well-known and understood. However, inflammasome-independent functions of NLRP3 may play various roles in different cell types.Here we show that NLRP3 can act to induce tissue fibrosis by enhancing TGF-β receptor signaling and fibrosis-associated markers (31, 32, 44). NLRP3 can also regulate the translocation of intestinal microorganisms and enhance endothelial cell damage by increasing the expression of selectins, which promote neutrophil recruitment and thereby NETosis and granule secretion.

However, many of the cell-specific functions attributed to NLRP3 are inconsistent with the expression of its transcripts in various tissues and/or cell types, such as lung and kidney epithelial cells. It is likely that many of these conflicting results are due to problematic *in vitro* tools, such as immortalized cell lines of untested nature or, in the case of primary cells, possible immune cell contamination. Thus, the use of tools to delete or induce NLRP3 or its related molecules in specific cell types, e.g., knockout, knockin, should be better explored so that new therapeutic alternatives can be created and even expand beyond merely immune-mediated diseases.

## Author contributions

All authors contributed equally to this review. All authors contributed to the article and approved the submitted version.
